# Promise and Peril of a Genotype‐First Approach to Mendelian Cardiovascular Disease

**DOI:** 10.1161/JAHA.123.033557

**Published:** 2024-10-18

**Authors:** Babken Asatryan, Brittney Murray, Rafik Tadros, Marina Rieder, Ravi A. Shah, Ghaith Sharaf Dabbagh, Andrew P. Landstrom, Stephan Dobner, Patricia B. Munroe, Christopher M. Haggerty, Argelia Medeiros‐Domingo, Anjali T. Owens, Iftikhar J. Kullo, Christopher Semsarian, Tobias Reichlin, Andreas S. Barth, Dan M. Roden, Cynthia A. James, James S. Ware, C. Anwar A. Chahal, the Genotype‐First Approach Investigators

**Affiliations:** ^1^ Division of Cardiology, Department of Medicine Johns Hopkins University School of Medicine Baltimore MD USA; ^2^ Department of Cardiology Inselspital, Bern University Hospital, University of Bern Bern Switzerland; ^3^ Cardiovascular Genetics Centre Montréal Heart Institute Montréal Québec Canada; ^4^ Royal Brompton Hospital, Guy’s and St Thomas’ NHS Foundation Trust London United Kingdom; ^5^ Center for Inherited Cardiovascular Diseases WellSpan Health Lancaster PA USA; ^6^ Division of Cardiovascular Medicine University of Michigan Ann Arbor MI USA; ^7^ Division of Cardiology, Department of Pediatrics, and Department of Cell Biology Duke University School of Medicine Durham NC USA; ^8^ NIHR Barts Biomedical Research Centre William Harvey Research Institute, Queen Mary University of London London United Kingdom; ^9^ Department of Translational Data Science and Informatics Heart Institute, Geisinger Danville PA USA; ^10^ Swiss DNAlysis Dübendorf Switzerland; ^11^ Center for Inherited Cardiovascular Disease, Cardiovascular Division University of Pennsylvania Perelman School of Medicine Philadelphia PA USA; ^12^ Department of Cardiovascular Medicine Mayo Clinic Rochester MN USA; ^13^ Agnes Ginges Centre for Molecular Cardiology at Centenary Institute, The University of Sydney Sydney New South Wales Australia; ^14^ Faculty of Medicine and Health The University of Sydney Sydney New South Wales Australia; ^15^ Department of Cardiology Royal Prince Alfred Hospital Sydney New South Wales Australia; ^16^ Department of Medicine, Pharmacology, and Biomedical Informatics Vanderbilt University Medical Center Nashville TN USA; ^17^ Program in Medical and Population Genetics Broad Institute of MIT and Harvard Cambridge MA USA; ^18^ National Heart and Lung Institute & MRC London Institute of Medical Sciences, Institute of Clinical Sciences, Faculty of Medicine, Imperial College London London United Kingdom; ^19^ Royal Brompton & Harefield Hospitals Guy’s and St. Thomas’ NHS Foundation Trust London United Kingdom; ^20^ Barts Heart Centre St Bartholomew’s Hospital, Barts Health NHS Trust London West Smithfield United Kingdom

**Keywords:** biobank, cardiac arrhythmia, cardiomyopathy, genetics, precision health, precision medicine sudden cardiac death, Genetics, Precision Medicine, Sudden Cardiac Death, Big Data and Data Standards

## Abstract

Precision medicine, which among other aspects includes an individual's genomic data in diagnosis and management, has become the standard‐of‐care for Mendelian cardiovascular disease (CVD). However, early identification and management of asymptomatic patients with potentially lethal and manageable Mendelian CVD through screening, which is the promise of precision health, remains an unsolved challenge. The reduced costs of genomic sequencing have enabled the creation of biobanks containing in‐depth genetic and health information, which have facilitated the understanding of genetic variation, penetrance, and expressivity, moving us closer to the genotype‐first screening of asymptomatic individuals for Mendelian CVD. This approach could transform health care by diagnostic refinement and facilitating prevention or therapeutic interventions. Yet, potential benefits must be weighed against the potential risks, which include evolving variant pathogenicity assertion or identification of variants with low disease penetrance; costly, stressful, and inappropriate diagnostic evaluations; negative psychological impact; disqualification for employment or of competitive sports; and denial of insurance. Furthermore, the natural history of Mendelian CVD is often unpredictable, making identification of those who will benefit from preventive measures a priority. Currently, there is insufficient evidence that population‐based genetic screening for Mendelian CVD can reduce adverse outcomes at a reasonable cost to an extent that outweighs the harms of true‐positive and false‐positive results. Besides technical, clinical, and financial burdens, ethical and legal aspects pose unprecedented challenges. This review highlights key developments in the field of genotype‐first approaches to Mendelian CVD and summarizes challenges with potential solutions that can pave the way for implementing this approach for clinical care.

**Nonstandard Abbreviations and Acronyms**
FH: familial hypercholesterolemia
P/LP: pathogenic/likely pathogenic
PRS: polygenic risk score
WES: whole‐exome sequencing

Excellence is never an accident. It is always the result of high intention, sincere effort, and intelligent execution; it represents the wise choice of many alternatives; choice, not chance, determines your destiny. Aristotle

Two decades have passed since the Human Genome Project^1^ released the first draft of the human genome. The subsequent rapid development of next‐generation sequencing in clinical practice has led to decreased costs of genetic sequencing and diversity of sequenced genomes, with a shift in the clinical care of patients with genetic diseases.^2^ Accessibility to genetic testing, through expanding clinical programs and consumer‐initiated testing, has resulted in more queries by patients and clinicians to identify genetic cause. Furthermore, with some conditions, a genetic test can refute or confirm subtypes, as well as aid with ontology; for example, genetic testing for a hypertrophic phenotype may identify hereditary transthyretin amyloid and exclude Fabry disease, as well as known sarcomere causes. The sentiment of close approximation of predictive genomics with health care is reflected in the National Human Genome Research Institute strategic vision published in 2020.^3^ Poised to harness resources and intellectual energy to synergize science and clinical care, the cardiovascular community has led discoveries and implementation of evidence‐based medicine, which aligns with the goals of precision medicine.^4^ Precision medicine, an innovative approach to disease prevention and management tailored to an individual's genetic background, environmental influences, and lifestyle, has already led to key discoveries in the area of diagnosis, prognostication,^5^ and Food and Drug Administration‐approved treatments that alter Mendelian cardiovascular disease (CVD) progression and improve outcomes.^6^, ^7^, ^8^

For some inherited CVD, such as Fabry disease, dystrophin‐deficient and transthyretin amyloid cardiomyopathies, and familial hypercholesterolemia (FH), precision medicine is already a reality thanks to disease‐modifying therapies. For others, such as long‐QT syndrome (LQTS) and hypertrophic cardiomyopathy (HCM), mechanism‐based therapies are now available. In arrhythmogenic and dilated cardiomyopathies (DCM), growing knowledge suggests an important role of genetic testing for prognostication, particularly on the risks of progressive heart failure (HF) and sudden cardiac death (SCD).^9^, ^10^ To benefit from these treatments and prevent disease complications, timely diagnosis and family screening are imperative. Nonetheless, many patients with these conditions present with complex and heterogeneous phenotypes overlapping with other entities, which often leads to a significant delay in diagnosis. Moreover, a considerable proportion of patients with Mendelian CVD present at an advanced disease stage not amenable to therapy, or with SCD as a sentinel manifestation of disease and diagnosis postmortem after a decades long silent course,^11^ highlighting the urgent need for methods to identify those at risk for Mendelian CVD and implement measures toward prevention of such devastating outcomes.

Over the past few years, the concept of a genotype‐first approach was formed, a preventative strategy that focuses on identifying underlying genetic causes of diseases before the presentation of clinical symptoms, in contrast to the phenotype‐driven identification of those who often already have a phenotypically robust disease (frequently in its advanced stages), who may benefit from genetic testing. A genotype‐first approach has emerged as a powerful tool to study the genetic epidemiology and clinical penetrance of Mendelian diseases in genetic biorepositories with phenotypic characterization. Furthermore, this approach provides the means to explore genetic epidemiology, pleiotropy, and variable expressivity of genetic conditions. Precision health, a health care approach that combines traditional medical practices with technological advancements such as genomics and big data analytics to provide more precise and effective health care, is emerging as a novel methodology that might help address multiple gaps in the clinical care of patients with Mendelian CVD. Unlike precision medicine, which addresses established diseases, precision health focuses on genetic predisposition, removing the bias introduced by phenotype. Because many health care systems are increasingly prioritizing value‐based health care and a drive for population health,^12^ precision health has a role, and its most promising role may be in Mendelian CVD.

The reduced costs of genomic sequencing, advances in our understanding of genotype–phenotype associations, and gene‐ and mechanism‐based interventions have paved the path for the application of genetic screening for Mendelian CVD to inform population health. The effect size of pathogenic variants is usually markedly higher for Mendelian CVD (Figure 1), such as *LMNA* for cardiac laminopathy,^13^ *LDLR* for familial hypercholesterolemia,^14^ *GCK* for diabetes,^15^ and *KCNH2* for LQTS type 2 (LQT2).^16^ In contrast, common CVDs, such as coronary artery disease (CAD) and atrial fibrillation (AF), usually have multifactorial inheritance, with several genetic loci as well as environmental factors contributing to the disorder, although Mendelian forms of disease are increasingly recognized. Herein, we discuss the evidence for and against using a genotype‐first approach to diagnose Mendelian CVD and how identifying at‐risk individuals in this fashion may inform surveillance and management plans. There is also enthusiasm for identifying high‐risk individuals using combined approaches of Mendelian with polygenic risk scores (PRS) and how these may also modify Mendelian disease risk,^17^ as discussed briefly below. However, the major focus of this review is on population screening for Mendelian disease, and PRS as a means to predict development of common CVDs, such as AF and CAD, is beyond the scope of this review. Terms commonly used in this review are defined in the Table.

**Figure 1:**
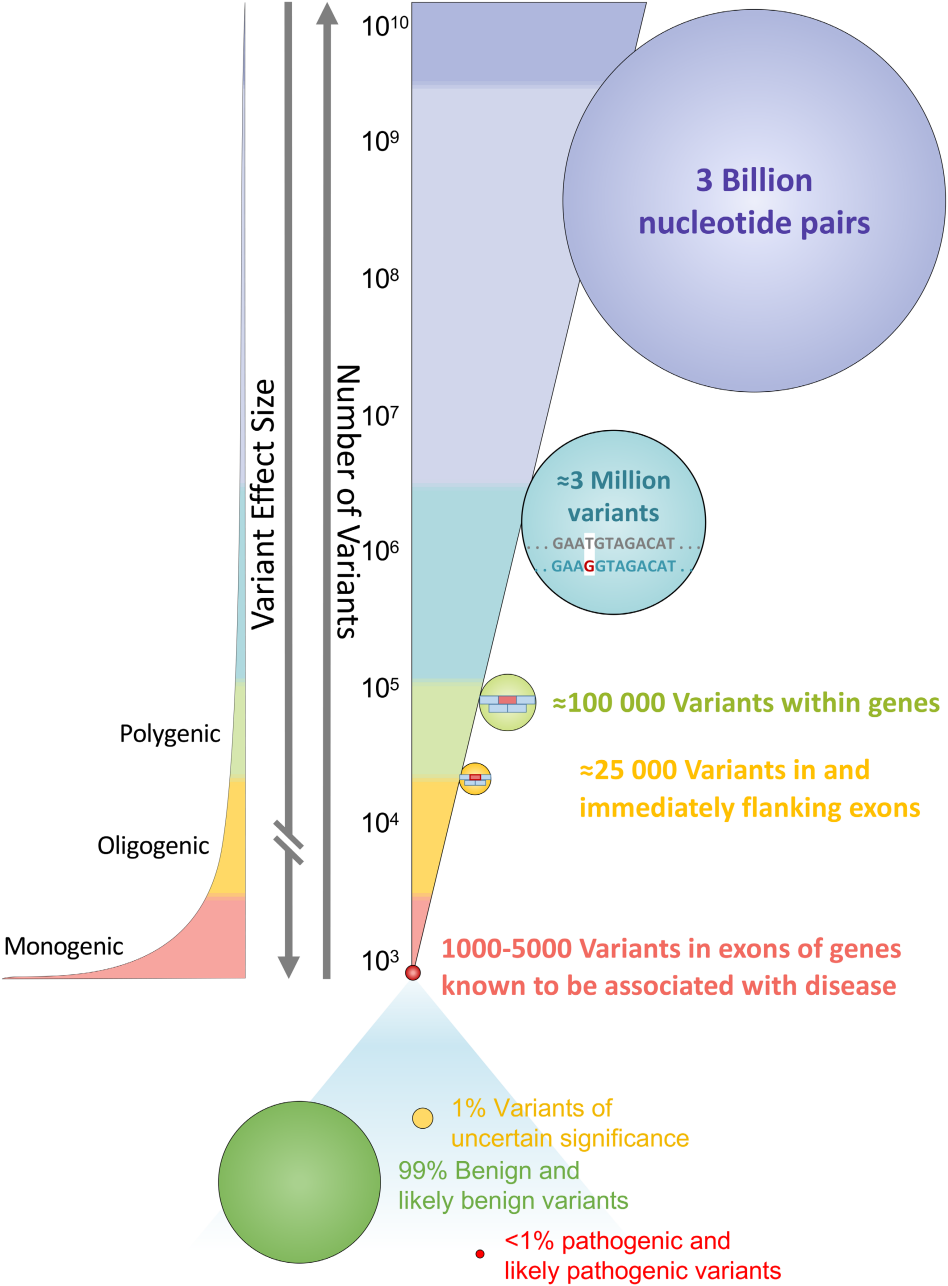
Variant allele frequency and effect size associations. The figure highlights that among the 3 billion nucleotide pairs in an individual's genome, nearly 3 million variants are hosted, of which ≈100 000 are located within genes and 1000 to 5000 in exons of genes known to be associated with disease. However, of those, <1% are pathogenic, disease‐causing variants, indicating that a rare variant does not always imply a pathogenic variant. On the other hand (left side scale), a higher allele frequency indicates a higher likelihood of the variant being a benign variation and thus less likely to cause a Mendelian disease.

**Table 1:** Common Genetic and Genomic Terms Used in this Review*

Term	Definition
Carrier screening	Carrier screening involves testing to see if a person carries a genetic variation (allele) associated with a specific disease or trait.
Complex disease	A complex disease (or condition), when discussed in the context of genetics, reflects a disorder that results from the contributions of multiple genomic variants and genes in conjunction with significant influences of the physical and social environment. For this reason, complex diseases are also called multifactorial diseases. This stands in contrast to a simple genetic disease that is more directly caused by variants in a single gene.
Gene–environment interaction	Gene–environment interaction refers to the interplay of genes (and more broadly genome function) and the physical and social environment. These interactions influence the expression of phenotypes.
Genetic counseling	Genetic counseling refers to guidance relating to genetic disorders that a specialized health care professional (genetic counselor) provides to an individual or family. A genetic counselor might provide information about how a genetic condition could affect an individual or family and/or interpret genetic test results, conveys information to address the concerns of the individual or family, helps them make an informed decision about their medical situation, and provides psychological counseling to help them adapt to their condition or risk.
Genetic discrimination	Genetic discrimination refers to the unequal treatment of individuals based on an aspect of their genetic code or genome, such as the risk for genetic disorder. Genetic discrimination can involve such genomic information being used against individuals in a variety of circumstances, such as employment, health or disability, insurance status, education, or health care.
Genotype‐first approach†	Genotype‐first approach is a diagnostic approach that is directed to tracking and prevention of disease, which uses genomic data to identify subjects at genetic risk, irrespective of the phenotype.
Mendelian disease/inheritance	Mendelian inheritance refers to certain patterns of how traits are passed from parents to offspring. These general patterns were established by the Austrian monk Gregor Mendel, who performed thousands of experiments with pea plants in the 19th century. Mendel's discoveries of how traits (such as color and shape) are passed down from one generation to the next introduced the concept of dominant and recessive modes of inheritance.
Newborn screening	Newborn screening is a set of laboratory tests performed on newborn babies to detect a set of known genetic diseases. In the United States, newborn screening is mandatory for a defined set of genetic diseases, although the exact set differs from state to state. Newborn screening tests focus on conditions for which early diagnosis is important to treating or preventing disease.
Penetrance	Penetrance is the extent to which a particular genotype or set of genotypes is expressed in the phenotypes of individuals carrying it, measured by the proportion of carriers showing the characteristic phenotype.
Phenotype	Phenotype refers to an individual's observable traits, such as height, eye color and blood type. A person's phenotype is determined by both their genomic makeup (genotype) and environmental factors.
Phenotype‐first approach†	Phenotype‐first approach is the established traditional model of health care in genetic medicine that focuses on the diagnosis and treatment of disease in those with robust clinical phenotype.
Polygenic risk score	A polygenic risk score uses genomic information alone to assess a person's chances of having or developing a particular medical condition. A person's polygenic risk score is a statistical calculation based on the presence or absence of multiple genomic variants, without taking environmental or other factors into account.
Polygenic trait	A polygenic trait is a characteristic that is influenced by ≥2 genes. Because multiple genes are involved, polygenic traits do not follow the patterns of Mendelian inheritance. Many polygenic traits are also influenced by the environment and are called multifactorial.
Population genetics	Population genomics is the large‐scale application of genomic technologies to study populations of individuals.
Precision medicine	Precision medicine (generally considered analogous to personalized medicine or individualized medicine) is an innovative approach that uses information about an individual's genomic, environmental, and lifestyle information to guide decisions related to their medical management. The goal of precision medicine is to provide a more precise approach for the prevention, diagnosis, and treatment of disease.
Risk	Risk, as related to genetics, refers to the probability that an individual will be affected by a particular heritable or genetic disorder. Both a person's genome and environmental exposures can influence risk. An individual's risk may be higher because they inherit a genetic variant (or allele) in 1 gene or a combination of many variants in different genes that increases susceptibility to or overtly causes a disorder.
Secondary genomic finding	A secondary genomic finding refers to a genomic variant, found through the analysis of a person's genome, that is of potential medical value yet is unrelated to the initial reason for examining the person's genome. In certain cases, a secondary genomic finding might offer clinicians the chance to identify a previously unrecognized risk for disease that could change the medical management of that patient and potentially prevent or more effectively treat the disease.
Susceptibility	Susceptibility, as related to genetics, refers to the state of being predisposed to, or sensitive to, developing a certain disease. An individual's disease susceptibility is influenced by a combination of genetic and environmental factors.
Variant of uncertain significance	When analysis of a patient's genome identifies a variant, but it is unclear whether that variant is actually connected to a health condition, the finding is called a variant of uncertain significance. In many cases, these variants are so rare in the population that little information is available about them. Typically, more information is required to determine if the variant is disease related. Such information may include more extensive population data, functional studies, and tracing the variant in other family members who have or do not have the same health condition.

* Definitions are based on *Talking Glossary of Genomic and Genetic Terms* of the National Human Genome Research Institute (https://www.genome.gov/genetics‐glossary), unless indicated otherwise.

^†^ Defined for this review in the context of the general trends in the field.

## LARGE‐SCALE GENETIC BIOBANKS OFFER A NEW PROSPECT INTO CVD GENETICS

To support scientific discovery and enable a cost‐effective and hypothesis‐neutral approach to cohort generation, different research networks have initiated large‐scale population‐based prospective studies with collection and analysis of biological samples (Table S1). Biobanks such as BioVU at Vanderbilt, Intermountain Healthcare's–Precision Medicine Biobank and HerediGene Project, UK Biobank, Mayo Tapestry, Helix Research Network (United States), MyCode at Geisinger, Montreal Heart Institute Biobank, Million Veteran Program (Veterans Affairs System, United States), BioMe Biobank Program at Icahn School of Medicine, National Institutes of Health All of Us, the Gene Health Project at WellSpan Health (part of the Helix Research Network), Estonian Biobank, Auria Biobank (Finland), FinnGen (Finland), deCODE Genetics (Iceland), Biobank Graz (Austria), BioBank Japan, China Kadoorie Biobank, Pakistan Genomic Resource Biobank, Qatar Biobank, and others provide high‐dimensional data with large sample sizes, allowing gains in statistical power toward identification of novel genetic findings. Besides furthering the knowledge of cardiac physiology,^18^ leveraging biobanks has enabled the identification of multiple previously unappreciated genes with a causal role in human congenital heart disease,^19^ and provided deeper insights into the genetic basis of AF,^20^ CAD,^21^ inherited arrhythmias,^16^, ^22^ cardiomyopathies,^23^, ^24^ cardiometabolic disorders,^25^ and HF.^26^ Biobanks have been successfully used by the biopharmaceutical industry to improve success in drug discovery for human diseases,^27^ using Mendelian randomization as a technique to test causal inference (eg, IL6R as a proxy for tocilizumab).^28^ Biobanks were also used by multiple research groups to develop and validate PRS. In terms of the genotype‐first approach, biobanks are key to assessing variant pathogenicity and estimating penetrance, particularly when integrated phenotype data are available. Furthermore, multiancestry, genome‐wide genetic discovery studies provided substantial improvements in fine‐mapping functional variants and portability of polygenic prediction.^29^ Most importantly, these research resources continuously expand the knowledge on human gene variation beyond patient cohorts. However, most if not all biobanks are not true representations of the general population due to ascertainment and healthy volunteer biases, among others. Notably, some biobanks focus on health and others (usually health system‐based systems) on disease.

## GENOTYPE‐FIRST APPROACH TO MENDELIAN CVD: EVIDENCE FROM GENOMIC BIOBANKS

The genotype‐first approach has enabled revisiting the estimates of genetic predisposition to Mendelian CVD at a population level.^24^, ^30^ Several studies have found a prevalence of ultrarare, possibly disease‐associated, variants greater than the estimated prevalence of clinical disease caused by those variants.^24^, ^31^ The summaries below highlight the great potential of a genotype‐first approach to Mendelian CVD.

### Whole‐Exome Sequencing Screening for a Broad Range of Mendelian CVD

Data from the Catheterization Genetics registry showed that 4.5% (389 out of 8574) of individuals harboring pathogenic or likely pathogenic (P/LP) variants in cardiac‐associated genes, identified with whole‐exome sequencing (WES), were predicted to have at least 1 Mendelian CVD (cardiomyopathies, arrhythmias, connective tissue disorders/aortopathies, and FH).^32^ Additionally, up to 149 (1.7%) patients carried both a P/LP variant and demonstrated features suggesting phenotypic expression of the relevant disease; however, only 35% (52 out of 149) of individuals had been given the relevant clinical diagnosis. The most prevalent diagnosis missed was familial *TTR* (transthyretin) amyloidosis, followed by various forms of cardiomyopathies (genes implicated: *MYH7*, *MYBPC3*, *TNNI3*, *TNNT2*, *TTN*, *BAG3*, *LMNA*, *DES*, and *FLNC*), and heterozygous FH (gene implicated: *LDLR*).^32^ Notably, this in‐hospital cohort appears to be enriched with Mendelian CVD phenotypes as compared with other biobanks and the general population, as described in the following sections.

### Familial Hypercholesterolemia

Analyzing *LDLR*, *APOB*, and *PCSK9* for P/LP variants is designated by the Centers for Disease Control and Prevention as a tier 1 genomic application, with strong evidence to support population testing for early detection and intervention. A landmark study from the Geisinger Health System published in 2016 showed that genomic screening can prompt the diagnosis of patients with FH.^33^ Following WES‐based identification of those with a genetic cause of FH, the authors assessed the electronic health records (EHRs) for diagnosis of FH and appropriate pharmacotherapy. The estimated FH prevalence was 1 out of 256 in unselected participants from the health care system and 1 out of 118 in participants recruited via the cardiac catheterization laboratory. FH variant carriers had a significantly increased risk of CAD, but only 24% of them met EHR‐based presequencing criteria for probable or definite FH diagnosis. Active statin use was identified in 58% of carriers; 46% of statin‐treated carriers had a low‐density lipoprotein (LDL) cholesterol of <100 mg/dL.^33^ In another study investigating whether a genetic variant for FH alters the risk of atherosclerotic CVD incremental to serum lipid levels, Trinder et al performed a genetic‐association study using the UK Biobank.^34^ Genotyping array and WES data from the UK Biobank cohort were used to identify individuals with monogenic (*LDLR*, *APOB*, and *PCSK9*; n=277) or polygenic hypercholesterolemia (LDL cholesterol PRS >95% on 223 single‐nucleotide variants in the entire cohort, n=2379). A monogenic FH‐associated variant was identified in 1 in 176 individuals from the UK Biobank and was associated with elevated levels of LDL cholesterol and 3‐fold increased risk for an atherosclerotic CVD event at ≤55 years of age (6.1% versus 2.0%, *P*<0.001). Furthermore, among participants with comparable levels of LDL cholesterol, both monogenic (hazard ratio [HR], 1.93 [95% CI, 1.34–2.77]; *P*<0.001) and polygenic hypercholesterolemia (HR, 1.26 [95% CI, 1.03–1.55]; *P*=0.03) were significantly associated with an increased risk of CVD events compared with the risk of such events in individuals with hypercholesterolemia without an identified genetic cause. The Geisinger study recruited a more disease‐enriched population given it is health care system‐based, as compared with the UK Biobank, a middle‐aged general population (40–69 years of age at recruitment) with volunteer bias, despite which results were remarkably similar.

### Dilated Cardiomyopathy

Multiple research groups have studied the impact of truncating variants in the *TTN* (titin) gene (TTNtv) in individuals without known heart disease at the population level. Using machine‐based analysis of high‐resolution cardiac scans, Schafer et al found that TTNtv are associated with eccentric cardiac remodeling in healthy humans.^35^ To evaluate the clinical importance of identifying a TTNtv in an asymptomatic individual with respect to relative and absolute risks of future cardiovascular disease, Pirucello et al analyzed gene sequencing data in 45 346 UK Biobank participants without a prior diagnosis of HF, AF, or CAD.^36^ Over a median follow‐up of 6.9 years, the composite primary end point (physician diagnosis code of DCM, HF, AF, and all‐cause mortality) occurred in 9.3% of TTNtv carriers and 4.6% of noncarriers (adjusted HR, 2.2 [95% CI, 1.3–3.5]; *P*=0.002). However, most participants with TTNtv remained free of a new CVD in a prospective follow‐up, indicating that superimposed clinical or environmental stressors, such as chemotherapy, alcohol use, and pregnancy, may unmask disease among those genetically predisposed by a TTNtv. In another genotype‐first approach study, Haggerty et al leveraged the WES data of >71 000 individuals (Geisinger MyCode Community Health Initiative and PennMedicine BioBank) to identify individuals with TTNtv.^37^ They demonstrated that individuals of European ancestry with a TTNtv in a highly expressed exon have an increase in the population with a low left ventricular ejection fraction and an increased odds of DCM. Associations with arrhythmias, including AF, were observed even when controlling for a cardiomyopathy (CMP) diagnosis, suggesting that identification of such variants may help advance clinical risk stratification. Another recent study by Shah et al investigated the clinical and subclinical penetrance of ultrarare, possibly disease‐associated variants in DCM‐associated genes in the UK Biobank.^24^ Analyzing WES and using advanced ECG and cardiac magnetic resonance imaging data from >18 000 individuals, they demonstrated that approximately 1 in 6 individuals with ultrarare variants in DCM‐related genes and no clinical diagnosis of DCM exhibit a subclinical DCM phenotype or associated features based on ECG or cardiac magnetic resonance imaging.^24^ This approach uncovered a wide spectrum of DCM phenotypic heterogeneity and disease severity, showing that approximately 16 arrhythmias in the absence of substantial ventricular dilation/dysfunction are the most common manifestation of ultrarare variants in DCM‐associated genes.^24^ Building on this early work, in a subsequent study leveraging the UK Biobank WES data from >200 000 individuals, it has been demonstrated that individuals with ultrarare variants in cardiomyopathy‐related genes are at higher risk for all‐cause death irrespective of ultimate phenotype (HR, 1.07 [95% CI, 1.02–1.13]; *P*=0.01) and for being diagnosed with cardiomyopathy later in life (HR, 2.38 [95% CI, 1.98–2.86]; *P*<0.0001), as well as for developing the composite outcome of a diagnosis of cardiomyopathy, HF, arrhythmia, stroke, and death.^38^ The penetrance assessments by Shah et al are consistent with a recent study by McGurk et al, which estimated penetrance through large‐scale analyses of patients referred for diagnostic sequencing for DCM, using a cross‐sectional approach comparing allele frequencies against reference populations.^39^ The aggregate penetrance of P/LP variants in TTNtv was 9.8% (8.0%–12.1%) and in all strong and definitive evidence DCM‐associated genes combined 11.3% (9.3%–13.6%).

In another report, Carruth et al studied the EHR phenotype in individuals with loss‐of‐function *FLNC* variants (*FLNC*_LOF_) at the MyCode cohort, and found that at least 9% of these patients showed evidence of penetrant disease.^40^ Individuals with *FLNC*_LOF_ variants had a nearly 5‐fold increased risk for DCM, 3‐fold higher risk for supraventricular tachycardia, and 4‐fold higher risk for left‐dominant arrhythmogenic cardiomyopathy.^40^ This contrasts with the study by McGurk et al, in which there were no cases of inherited cardiomyopathy among 50 P/LP *FLNC* truncation variant carriers in the UK Biobank.^41^ The limited sensitivity of EHRs and the depletion of the UK Biobank of the most severe and penetrant cases due to healthy volunteer bias, and recruitment of participants 40 to 69 years of age may be a contributing factor to this difference between studies. However, considering these observations, care should be taken in assessing the evidence behind each gene before making conclusions on genotype‐first screening.

### AF as Part of the Cardiomyopathic Phenotype Spectrum

Another gap that can be filled at least in part by the genotype‐first approach is the screening and identification of the genetic basis of AF. Genetic testing is increasingly used in AF, and international consensus suggests it might be useful (class IIB recommendation) in selected patients with AF.^42^ A recent study with whole‐genome sequencing in 1293 patients (72% men; median age, 56 [48–61] years at enrollment and 50 [41–56] years at AF diagnosis) with early‐onset AF (<65 years of age) identified ≈10% to carry P/LP in cardiomyopathy (mainly) or inherited arrhythmia‐associated genes.^43^ The most commonly implicated genes were *TTN* (n=38), *MYH7* (n = 18), *MYH6* (n=10), *LMNA* (n=9), and *KCNQ1* (n=8). Other studies confirmed that TTNtv carriers in the general population are at significantly higher risk for AF (in addition to DCM).^44^ In a study using the UK Biobank, Choi et al sought to determine the contribution of rare and common genetic variation to AF risk in a general population cohort 40 to 69 years of age.^45^ The study included 1546 AF cases (mean age at AF onset of 62.6±7.3 years; 33.7% women) and 41 593 controls (54.9% women). In an analysis of 9099 genes with sufficient numbers of loss‐of‐function variant carriers, a significant association between AF and rare loss‐of‐function variants was observed in a single gene, *TTN* (odds ratio [OR], 2.71; *P*=2.50×10^−8^), and this association was stronger (OR, 6.15; *P*=3.26×10^−14^) when restricting to loss‐of‐function variants (*TTN*_*LOF*_) located in exons highly expressed in cardiac tissue. Overall, 0.44% of individuals carried *TTN*_*LOF*_, of whom 14% had AF (mean age at AF onset of 62.6±7.6 years; 41.2% women). Remarkably, AF PRS showed striking differences in the prevalence of AF among *TTN*_*LOF*_ variant carriers (OR per SD, 1.79); *TTN*_LOF_ variant carriers in the lowest tertile of AF PRS had an AF prevalence of only 6.7% compared with 21.5% in the highest tertile. Thus, PRS results in an additive risk that further increases the likelihood of AF in *TTN*_LOF_ mutation carriers. This indicates an opportunity for timely identification of at‐risk subjects and, upon emergence of sufficient supporting data, early initiation of AF control and anticoagulation to prevent HF and stroke, respectively. Furthering the link between AF and LV remodeling and function, Schiabor Barrett et al demonstrated that one‐third of individuals with *TTNtv* and early AF will develop DCM at follow‐up.^46^ Similarly, Shoemaker et al showed that carrying a disease‐associated variant in cardiomyopathy‐associated genes was associated with a significantly higher risk of mortality when AF was diagnosed at a younger age,^47^ indicating the potential usefulness of using AF as a biomarker of progressive disease in *TTNtv* (and possibly other cardiomyopathy gene P/LP) carriers.

### Hypertrophic Cardiomyopathy

HCM is the most common genetic cardiomyopathy, typically caused by P/LP variants in sarcomere genes, and usually manifests in adulthood. Notably, previous studies have shown that pathogenic sarcomere variants known to cause HCM are unexpectedly common in the general population.^31^ In 2012, Seidman and colleagues reported a relatively high frequency of disease‐causing variants in a general community‐based study. In that investigation, 3600 participants of both sexes (30–84 years of age) were studied, including 1637 unrelated subjects in the offspring cohort of the Framingham Heart Study and 1963 unrelated subjects from the Jackson Heart Study cohort. Screening the principal 8 HCM‐causing sarcomere protein genes revealed at least 1 disease‐causing sarcomere variant in 0.6% of participants.^30^ A more recent study by de Marvao investigated the lifetime outcomes and cardiovascular phenotypes according to the presence of rare variants in sarcomere‐encoding genes among 200 584 middle‐aged adults.^48^ The prevalence of sarcomere P/LP variants for HCM was 0.25% (n=493; 1 in 407); these variants were associated with an increased risk of death or major adverse cardiac events compared with controls (HR, 1.69 [95% CI, 1.38–2.07]; *P*<0.001), mainly due to HF end points (HR, 4.23 [95% CI, 3.07–5.83]; *P*<0.001). Sarcomere HCM‐P/LP variants were associated with an asymmetric increase in left ventricular maximum wall thickness (10.9±2.7 mm versus 9.4±1.6 mm, *P*<0.001), but hypertrophy (≥13 mm) was only present in 18.4% (9 out of 49 [95% CI, 9%–32%]). Sarcomere HCM‐P/LP variants were still associated with HF after adjustment for wall thickness (HR, 6.74 [95% CI, 2.43–18.7]; *P*<0.001).^48^

### Inherited Arrhythmias

Genetic testing in LQTS is proving to be increasingly valuable in diagnosis, risk stratification, and management.^42^ The amazing progress in understanding disease mechanisms and the genotype–phenotype correlations have paved the way for gene‐specific management in patients with LQTS.^42^ In LQTS, the identification of a P/LP variant in *KCNQ1*, *KCNH2*, or *SCN5A*, together with the duration of the corrected QT (QTc) are critical in assessing the risk of life‐threatening arrhythmias in asymptomatic subjects. Identification of a disease‐causing variant typically invokes initiation of β‐blockade, even in patients who are phenotype negative at the time^42^; this is particularly important in children for primary prevention of arrhythmias. However, until recently, the genetics of LQTS at the population level has not been investigated. To explore this area, Nauffal et al used UK Biobank and TOPMed data to assess monogenic and polygenic contributions to QTc prolongation in the population setting.^16^ They found that of individuals with QTc >480 ms, nearly one‐quarter carried either a monogenic ultrarare, potentially disease‐associated variant, or had a PRS in the top decile (3.4% monogenic, 21% top decile of PRS), indicating that population genetic screening alone can identify at least every fifth patient with QTc prolongation. Importantly, in multivariable analyses, carriers of rare, potentially LQTS‐associated variants in *KCNQ1* (average 30.0 ms [22.2–37.8]; *P*=6.0×10^−14^) or *KCNH2* (average 55.5 ms [39.2–71.9]; *P*=4.0×10^−11^) had an increase in their QTc compared with noncarriers, whereas the observed QTc difference was not significant in those with rare, potentially LQTS‐associated *SCN5A* variants (average 9.2 ms [−1.0 to 19.4]; *P*=0.08), indicating that the value of PRS varies by underlying genotype.^16^

Another study by Glazer et al assessed the pathogenicity of variants in 10 inherited arrhythmia genes and related clinical phenotypes in the eMERGE‐III (Electronic Medical Records and Genomics Phase III) cohort, a multicenter prospective cohort that included 21 846 participants without previous indication for cardiac genetic testing.^49^ Among them, 120 participants (0.6%) carried P/LP variants in inherited arrhythmia genes. Compared with noncarriers, carriers of P/LP variants in inherited arrhythmia genes had a significantly higher burden of arrhythmia phenotypes in their EHRs. Fifty‐four participants had variant results returned, of which 19 had inherited arrhythmia diagnoses (primarily LQTS). Notably, 12 of these 19 participants were diagnosed only after variant results were returned (0.05%). In vitro electrophysiological evaluation of 50 variants of uncertain significance (VUS) in HEK293 cells resulted in the reclassification of 3 to likely benign and 8 to P/LP. These results indicate that population genetic screening has the potential to identify previously undiagnosed patients with LQTS who may benefit from further investigation.

Although no population screening studies have evaluated the screening and diagnostic yield for catecholaminergic polymorphic ventricular tachycardia, genotype‐first screening may offer the potential to reduce SCD burden in patients with undiagnosed catecholaminergic polymorphic ventricular tachycardia. This becomes more imperative given that patients suffering catecholaminergic polymorphic ventricular tachycardia‐associated fatalities are mostly adolescents and young adults.

These findings highlight the underrecognition of Mendelian CVD in the clinical setting and represent a missed opportunity, which could potentially be addressed by genetic screening.^32^ The findings of multiple examples of identification of inherited CVD in a genotype‐first approach raise the pertinent question: Would a genotype‐first approach to Mendelian CVD in a population setting enable multidisciplinary care and minimize life‐threatening events in individuals at risk for these conditions?

## POTENTIAL BENEFITS OF A GENOTYPE‐FIRST APPROACH TO MENDELIAN CVD

Not only can genotype identify Mendelian CVD as described above, but solely phenotype‐based diagnoses can be incorrect.^50^ There is also evidence that the application of a genotype‐first approach changes real‐world practice patterns.^51^ Because the risk of SCD in many Mendelian CVDs can be reduced with sympathetic blockade, antiarrhythmic medications, anti‐HF medications, lipid‐lowering/‐modifying medications, and/or implantable cardioverter‐defibrillators, early intervention might be the key to better patient outcomes. A genotype‐first model that reliably identifies patients with genomic risk for Mendelian CVD may substantially contribute to reducing mortality and morbidity through timely initiation of early initiation of surveillance or therapy in high‐risk subjects. From a translational perspective, a genotype‐first approach would allow us to understand genetic variation and penetrance at their full scale, which are underinvestigated for most Mendelian diseases, where research has been largely focused on phenotype, linkage disequilibrium, and candidate gene approaches. It would also allow us to understand the role of genetic effect modifiers, expanding our understanding of genome‐environment interactions.

## CHALLENGES AND POTENTIAL SOLUTIONS

### Difficulties in Variant Interpretation

**Figure 2:**
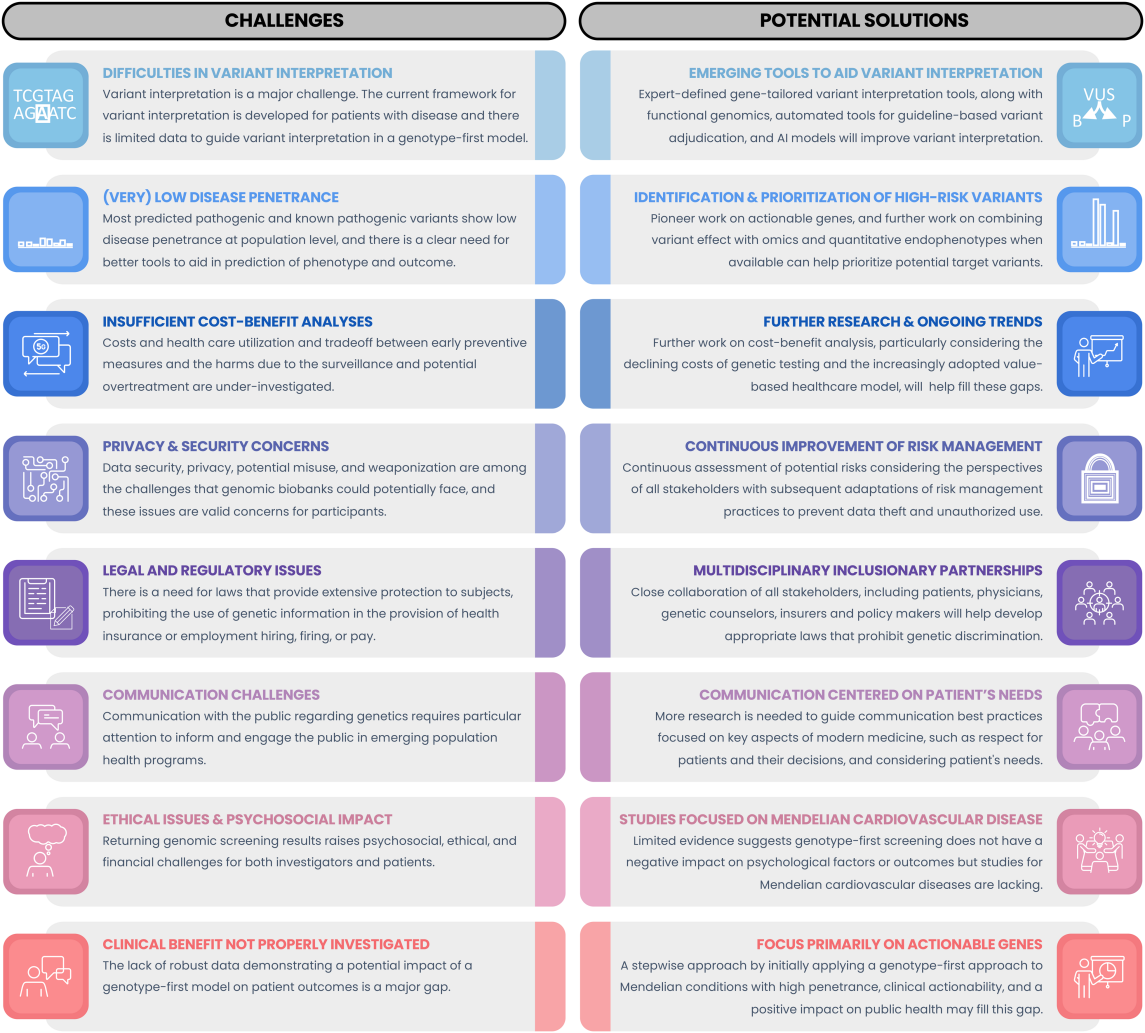
Challenges in applying a genotype‐first approach to Mendelian cardiovascular disease and potential solutions. AI indicates artificial intelligence.

**Figure 3:**
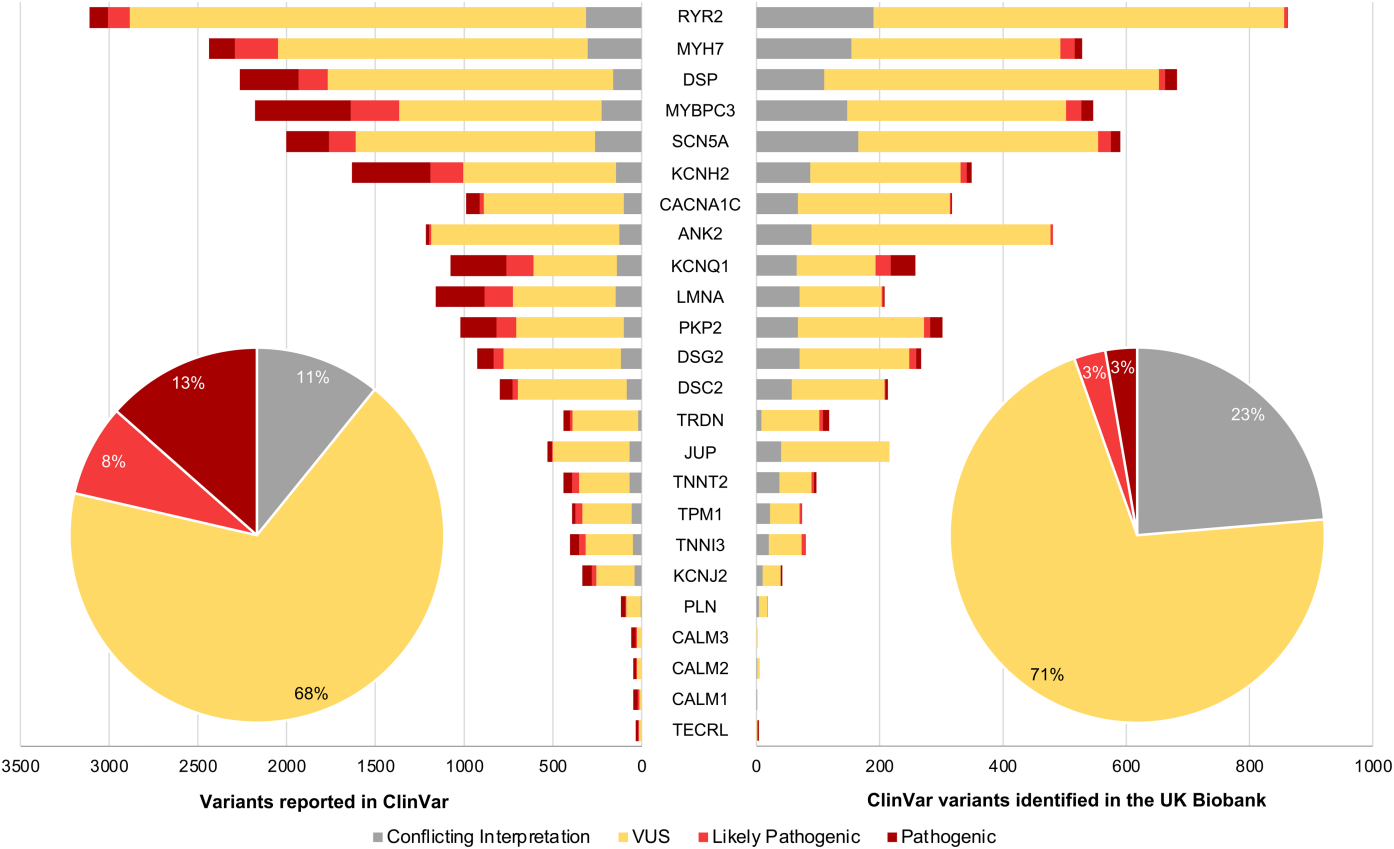
Variants reported in ClinVar (left panel) and the same variants identified in the UK Biobank (right panel) for a selected group of genes implicated in monogenic heart disease, classified based on their ClinVar‐listed American College of Medical Genetics and Genomics and the Association for Molecular Pathology classification. The left and right panels have different scales on the *x* axis. VUS indicates variants of uncertain significance.

Despite potential benefits, there remain significant challenges to implementing a genotype‐first population screening strategy (Figure 2). The major Achilles' heel of using next‐generation sequencing, particularly with WES/whole‐genome sequencing, is the determination of the pathogenicity of novel genetic variants. Most variants identified with genetic testing both in disease‐enriched patient cohorts and in the population settings are of uncertain significance (Figure 3). A VUS in a gene known to cause an inherited CVD can create a significant dilemma on its use in predictive testing and clinical diagnosis,^52^ which can trigger costly, stressful, and inappropriate diagnostic workups, and have negative implications for an individual.^53^ Importantly, gene variant interpretation is challenging even in the clinical setting, and assessment of VUS is laborious and prone to adjudicator disagreement and changes over time in light of new evidence. Therefore, these variants are often not included in the final clinical genetic test report to prevent unjustified anxiety and distress in the absence of medically actionable findings upon consent at pretest genetic counseling, but the policy might vary across jurisdictions.^54^ Such variants should be periodically reevaluated according to current knowledge, because reclassifications are common, with up to 50% of those implicated in Mendelian CVD being downgraded within a decade of the original report.^55^ Additionally, variant pathogenicity assertion in non‐White‐ancestry individuals is prone to high VUS rates, because allele frequencies are not established. Notably, cosegregation analysis is mostly not possible with research biobank data sets, and thus the population rate of de novo variants is not known, making variant interpretation even more challenging.

Ongoing efforts by expert panels to assess gene‐disease relationships (such as by Clinical Genome Resource),^56^, ^57^ and expert partnerships to develop universal standards and terminologies for evidence base, such as the Gene Curation Coalition,^58^ will improve the selection of genes of interest and variant assessment. International expert activities aimed at systematic and periodic evaluation of clinical actionability, potential interventions, and expected results of genetic testing using quantitative and semiquantitative scoring metrics, such as the Clinical Genomic Resource Actionability Working Group (https://actionability.clinicalgenome.org/), will allow the creation of repositories instrumental to the interpretation and reporting of genotype‐first approach findings.

A range of automatic tools have been developed to aid in variant interpretation, including tools that perform guidelines‐based pathogenicity assessment by implementing the American College of Medical Genetics and Genomics/Association for Molecular Pathology guidelines in a software/web tool,^59^ algorithms that convert the American College of Medical Genetics and Genomics/Association for Molecular Pathology recommendation in a probabilistic framework,^60^ or approaches that are data driven. The latter include machine‐learning models trained to distinguish pathogenic from benign variations,^61^, ^62^ or a combination of these methods.^63^ Although prior models have not been robustly rested in Mendelian CVD, there is a clear need to develop machine‐learning models that can help in variant prioritizing and interpretation.

Additionally, functional genomics methods are increasingly used to uncover patterns that aid in variant calling.^49^, ^64^ Combining functional imaging with artificial intelligence‐based methods of variant assessment can be a major step toward reducing the VUS burden.^65^

### Low‐Penetrance Variants and Uncertain Clinical Usefulness

The major challenge faced when considering a genotype‐first approach for clinical purposes is the lack of demonstrated benefit of a genotype‐first approach at the population level (ie, robust data from clinical trials testing this strategy assessing benefits, risks, and costs). Moreover, there are no clinical care pathways for patients with different genomic and clinical risk profiles. Another major difficulty is posed by the low clinical disease penetrance of Mendelian CVD in the general population settings as compared with disease‐enriched clinical cohorts and screened families.^24^, ^66^, ^67^, ^68^, ^69^, ^70^ Additional layers of unknowns include the appropriate course of surveillance in the absence of phenotype, as well as the risk profiles of patients with both phenotype and genotype identified through genomic screening, leaving the value of positive predictive value by genetic screening undetermined.^71^ Although it is unrealistic to provide effective genomic screening for all Mendelian CVDs at this point, a stepwise approach could be envisioned by initially applying a genotype‐first approach to conditions with high penetrance and clinical actionability, and a positive impact on public health may help pave the way for extending the care to a broader group of less penetrant conditions.^24^ For example, a pilot stage using this approach may focus on select high‐penetrance gene variants implicated in manageable Mendelian CVD, such as select *LMNA* and *SCN5A* variants. Another approach might be the 33 inherited CVD genes included in the American College of Medical Genetics (ACMG) list for reporting of secondary findings, or a selection of those genes that can serve as a reasonable starting point,^72^ as currently used at the All of Us program and the Geisinger Health System. This approach will also fill in gaps in the preliminary cost–benefit analysis and help increase awareness of mono−/oligogenic CVD with a potentially deadly outcome. To help further narrow the margin of risk into those with high‐confidence P/LP variants, specific techniques developed with the advancement of biobank knowledge, such as variant associations with quantitative endophenotypes for Mendelian CVD, can be used as additional evidence supporting pathogenicity (eg, LDL cholesterol for FH and QTc for LQTS).^73^ Overall, this approach will enable seeing beyond the tip of the iceberg of Mendelian CVD in the long term and expand the current landscape of precision medicine.

It is worth mentioning that the diagnostic weight of genetic testing at population setting is, at least for most known Mendelian CVDs, of screening value and not confirmatory unless there is a strong associated phenotype. This underscores the role of phenotyping, even in those with identified potentially disease‐associated variants. As such, the same *MYBPC3* variant identified in a patient with a robust HCM phenotype is a confirmation of a genetic disease (both in population and clinical settings), whereas its detection in a patient with normal cardiac magnetic resonance imaging or mild left ventricular hypertrophy in the absence of other causes only raises the modest possibility that the left ventricular hypertrophy may have a genetic basis (ie, potentially caused by the *MYBPC3* variant). Thus, phenotyping, including detection of subclinical/subdiagnostic phenotypes, using clinically validated and universally accepted tools (eg, the Schwartz score for LQTS, exercise stress testing for catecholaminergic polymorphic ventricular tachycardia (CPVT), serial imaging tests for HCM and DCM) are critical to the application of the genotype‐first approach.

### Defining Target Population

Children are particularly underrepresented and underinvestigated from a population genetics perspective, making a prediction on the usefulness of population screening and defining the age group to be screened difficult. For certain Mendelian CVDs, such as FH, treatment with statins starting in early childhood can reduce the risk of early myocardial infarctions, emphasizing the rationale for greater long‐term benefit with the initiation of FH treatment earlier rather than later in life.^74^ Although most other Mendelian CVDs have late onset in adulthood, almost all have less‐common childhood‐onset forms of disease. Furthermore, up to 5% of affected individuals with Mendelian CVDs have digenic or biallelic genotypes that manifest earlier in life, often with SCD^75^; thus, there is a survival bias as well as selection bias (healthy volunteer bias). There is also a considerable ancestry and genetic background bias in most biobanks, and only a few large biobanks, such as the Million Veteran Program and All of Us, include a substantial proportion of non‐White participants. Community engagement uniquely offers all stakeholders in human genetics and genomics an opportunity to address this underrepresentation in genomics medicine and research.^76^ Hence, the predominant knowledge generated concerns middle‐aged White participants, making generalizability to the entire human family poor, and significant efforts are underway to close the ancestry gap in current knowledge.

### Communication Challenges

Studies indicate that the majority of participants in a multiethnic biobank are interested in receiving genomic results for medically actionable conditions.^77^, ^78^ Similarly, there is strong evidence of high interest in the return of individual research results from potential and actual genomic research participants (an admittedly biased group).^79^ Successful implementation of a genotype‐first approach at the population level requires broad public participation, but precision public health involving population‐based DNA collection and sequencing has identified communication gaps, lack of transparency about data usage, and potential benefits for all parties involved.^80^ Studies have revealed some public concerns on the potential misuse of sequencing data for discriminatory purposes, for example, with regard to employment, privacy, or insurance, concerns that can be addressed at least in part by better communication practices.^81^ The Genetic Information Nondiscrimination Act was passed in 2008 to protect individuals undergoing genetic testing from having their genetic test results used to influence health insurance coverage or employment. There are considerable gaps in this law, however, including federal employees, life insurance, and long‐term disability insurance.^82^ Several other countries have government policies that protect patients from insurance exclusion while maintaining market sustainability.^83^ Since 2001, there has been a voluntary moratorium in the United Kingdom (agreed between the Association of British Insurers and the Department of Health) precluding the use of genetic information with strong predictive value for insurance purposes. However, there is extensive debate about whether the law enforcement mechanisms are adequate for its antidiscrimination intent.^84^ There remains, however, general support for the concept of genomic cardiovascular screening.^85^ A report from the All of Us research program indicated that 79% of individuals support and 54% are willing to participate in a genotype‐first screening.^86^ It is therefore apparent that more research is needed to guide communication best practices centered on key aspects of modern medical practice, such as clinical competence, respect for patients and their health care decisions, and maintaining the primacy of patient's needs, and should facilitate fine‐tuning of ethical aspects of the genotype‐first approach.

### Ethical Issues and Potential Psychosocial Impact

Returning genomic screening results raises psychosocial, ethical, and financial challenges for both investigators and patients. The latter, in particular, has been the focus of several studies. In a pilot trial, Christensen et al investigated the behavioral and psychological impact of genome sequencing in 100 apparently healthy, primary‐care participants and 100 cardiology participants.^87^ They randomized participants to receive a review of their family histories of disease, either alone or in addition to genetic screening for PRS for 8 cardiometabolic conditions. Although no differences were seen in the percentage of participants in the genetic screening and control arms, the odds of reporting a behavior change increased by 52% per high‐risk polygenic prediction. Mean anxiety and depression scores were similar between the arms, whereas mediation analysis suggested an indirect impact of genetic screening on health behaviors by causing positive psychosocial responses.^87^ Further studies from the eMERGE network^88^ have rigorously assessed the psychosocial impact of receiving actual genomic risk estimates related to other conditions. Assessments of different combinations of psychological factors and/or outcomes, such as perceived risk, motivation, self‐efficacy, confidence in disease‐related lifestyle changes, anxiety or worry responses, and psychosocial harm after the test results related to conditions such as type 2 diabetes or CAD, have found no adverse effects on receiving genomic risk information.^89^, ^90^, ^91^

Earlier studies identified self‐knowledge, reproductive planning, and practical benefits as the main factors determining the patient‐perceived value of genomics among those receiving genetic test results.^92^ These findings highlight the key role of genetic counseling services,^93^ which have evolved in the genomics era in response to increased demand across a broad range of contexts, while maintaining the core elements of patient‐centered genetic education, informed choice, and discussions of psychological implications of genomic information with individuals and families.^94^ Given that social determinants of health, such as low health literacy and lack of health insurance, influence the implementation of genomic medicine in low‐income populations in low‐resource settings, understanding such disparities will be necessary to better appreciate how genomic screening may fit within the context of health equity.^95^ Designing and implementation of genotype‐first screening programs should consider the challenges of participant perception and recruitment, and make sure individuals make informed decisions on their participation in such programs, with full comprehension of its objectives, main methodology, scope, potential clinical benefits, challenges, and the full breadth of potential negative implications. Genetic counselors are key to any model of genetic and genomic medicine and are the appropriate professionals to educate patients about genetic screening and their right to know and the right not to know while honoring the autonomy of individual informed decisions. Thus, consent, contact, engagement, and participation in genetic counseling must be considered before broader implementation of CVD genetic sequence results in health care.

### Cost‐Effectiveness Analysis

Costs and health care use and sharing of genomic results with family members are among the most important underinvestigated aspects of a genotype‐first approach to inherited CVD. Although economic value is a core principle in most deliberations about screening,^96^ there is only limited knowledge on cost estimates and tradeoffs between preventing avoidable Mendelian CVD burden via knowledge of its genetic basis and the harms due to the surveillance and potential overtreatment. A recent study by Christensen et al used a microsimulation model to simulate a US birth cohort of 3.7 million newborns and assessed the benefits, harms, and cost‐effectiveness of universal genetic screening for HCM at birth.^97^ The study found that newborn genetic screening would reduce HCM‐related deaths through age 20 years by 44 (95% uncertainty interval [UI], 10–103) but increase the number of children undergoing surveillance by 8127 (95% UI, 6308–9664). Compared with usual care, newborn genetic screening would cost $267 000 (95% UI, $106 000–$919000) per life‐year saved. Although $50 000 or $100 000 per quality‐adjusted life‐year are often used as benchmarks in analyses, there is no formal threshold for coverage decisions in the United States, and ethical arguments have been made to use much higher thresholds for the management of rare diseases.^98^ On the other hand, given the continuously declining cost of human genome sequencing, these cost estimates will decrease in the future. Combining the screening and surveillance for multiple Mendelian CVDs might be the key to a more cost‐effective model.

### Privacy and Security Issues

Data security, privacy, potential data misuse, and weaponization are among the diverse types of framing of risk biobanks could potentially face.^99^ Concerns on potential breaches in privacy or confidentiality of genetic information may deter people from participating in genetic screening for both clinical and research purposes.^100^ To ensure the responsible use of human biological samples and related data, designing a biobank should first include an analysis of the potential risks, considering the perspectives of all stakeholders such as institutional actors, participants, and representative organizations. Risk assessment should be a continuous adaptive process, with constant improvement of risk management practices to prevent data theft and unauthorized sharing of patient data in the digital era.

## CONCLUDING REMARKS AND FUTURE PERSPECTIVES

There is a rapidly growing potential to approach Mendelian CVD from the genotype‐first perspective using large‐scale population‐based biobanks integrating EHRs and genomic profiles. Implementing this nascent idea into the current health care model may have the potential to yield a substantial clinical benefit for a population subgroup at‐risk for Mendelian CVD, potentially translating into reduced morbidity and mortality. In particular, population‐level genetic screening can enhance disease prevention, refine diagnoses, improve prognostication, and enable deploying precision therapeutics for Mendelian CVD in at‐risk subjects who would not have been identified otherwise. However, the application techniques, including those specific to Mendelian CVD, are insufficiently studied due to the novelty of population‐based biobanks, insufficient validation of knowledge across biobanks, and lack of demonstration of improved outcomes and cost‐effectiveness. Surveillance and risk stratification of individuals at genomic risk both with and without phenotype are not informed by current clinical practice guidelines. Thus, the wide‐scale implementation must be tempered with a judicious assessment of the evidence. Additionally, while in the era of precision public health, everyone may be considered a possible participant in a genotype‐first model, whether to set screening eligibility simply by being born (newborn screening), by donating blood (biobanking), or through population screening, is unclear. Further work on validating the pathogenicity of variants, cost‐effectiveness analyses, development of proper surveillance methods, and understanding of how early measures can ameliorate the risk of developing disease or a specific outcome is required before population screening programs for Mendelian CVD can be designed. The influence of sex and lifestyle on the disease penetrance for most Mendelian CVDs at the population level remain underinvestigated, and focus on these aspects will be critical for evidence‐based counseling. Because increasing knowledge suggests that PRS can help to understand variability in penetrance, expressivity, and outcomes of Mendelian CVD, the creation of models that allow combining the powers of P/LP and PRS will help overcome burdens related to clinical phenotype and course in a genotype‐first screening approach. The development of standards for genomic screening should consider ancestry‐specific characteristics and address current knowledge gaps related to non‐White populations, who are vastly underrepresented in population genomic research. Importantly, policymakers and regulatory bodies proceeding with genetic screening programs should engage with all stakeholders, including lay public members. Profound ethical, legal, and policy considerations and issues related to the quality of life represent a particular challenge in a genotype‐first approach model; such a model should embrace ethical principles, such as autonomy, beneficence, fidelity, justice, and usefulness in line with the ideologies of modern medicine to ensure that individual rights are protected while clinical and research benefits are maximized. Enhanced partnerships between genetic and nongenetic providers of clinical medicine, academia, and public health are needed to fill in the gaps in the health care supply chain before implementing genotype‐first screening applications. When all these conditions are met, an evidence‐based action plan for a pilot trial of this approach, focusing on selected highly penetrant and manageable Mendelian CVDs, could help pave the way forward from precision medicine to precision health.

## Sources of Funding

Dr Asatryan is supported by the 2022 Research Fellowship for aspiring electrophysiologists from the Swiss Heart Rhythm Foundation, a postdoctoral research fellowship grant from the Gottfried and Julia Bangerter‐Rhyner‐Stiftung (Switzerland). Dr Rieder is funded by grants from the Gottfried und Julia Bangerter‐Rhyner‐Stiftung and the Bern University Hospital, Inselspital (Nachwuchsförderungs grant). Dr Semsarian is the recipient of a National Health and Medical Research Council Practitioner Fellowship (number 1154992) and is supported by a New South Wales Health Cardiovascular Disease Clinician Scientist grant.

## Disclosures

None.

## Supporting information

Table S1
