# Supplementary material for: Promise and Peril of a Genotype‐First Approach to Mendelian Cardiovascular Disease
Source: J Am Heart Assoc. 2024 Oct 18;13(21):e033557. doi: 10.1161/JAHA.123.033557 (PMC11935662; doi:10.1161/JAHA.123.033557)
Supplement: Supplementary file 1 — Table S1 [file JAH3-13-e033557-s001.pdf]

SUPPLEMENTAL MATERIAL

Table S1. Brief description of select biobanks with publicly available information.

Country	Year enrolled	Number of participants enrolled	Additional ongoing enrolment	Data and variables collected[*]				Funding	Additional information
				Biological measures	Surveys	Sample obtained	Genetics and other 'omics		
**USA**									
Million Veteran Program (USA)	2011	Target 1M	--	EHR	Self-completed<br>Lifestyle<br>Health	Blood	GWAS array<br>WES<br>WGS	USA DoH	• United States Department of Veterans Affairs
All of Us Research Program (USA)	2015	762k overall<br>523k completed initial steps<br>422k EHRs<br>541k+ biosamples	Further recruitment and completion of steps are ongoing	Anthropometrics<br>EHR	Self-completed questionnaires	Blood<br>Saliva	Genotyping<br>WES	NIH<br>Google Verily life Sciences	• Launched as 'Precision Medicine Initiative Cohort Program' and later renamed to 'All of Us Research Program'
BioVU, Vanderbilt University	2007	300k	Ongoing enrollment	EHR	--	Blood	WGS<br>Plasma biomarkers	NIH AHA NHMRC PCORI	• BioVU joined NHGRI's eMERGE (Electronic Medical Records and Genetics) Network
HerediGene, Intermountain Healthcare	2019	Target 500k<br>170k completed	Enrollment Paused	EHR	--	Blood<br>Saliva	Genotyping<br>WGS		• Scientific collaborator for sequencing the DNA: deCODE genetics in Reykjavík, Iceland and internally at intermountain.
Tapestry Study, Mayo Clinic	2020	100k	--	EHR	--	Blood<br>Saliva	WES		• Scientific collaborator for sequencing the DNA: Helix
Helix Research Network	2020	450k	Ongoing enrollment, goal 2M	EHR	--	Blood<br>Saliva	Exome+<br>WES on WGS backbone		• Gene-first in enriched populations of patients within healthcare systems who are more likely to have disease
MyCode Community Health Initiative, Geisinger	2007	300k	--	EHR	--	Blood<br>Saliva	WES	Robert Wood Johnson Foundation; Commonwealth of Pennsylvania Department of Community and Economic Development; Frontier Communications; The Horace W. Goldsmith Foundation; and Mericle Foundation	• Scientific collaborator for sequencing the DNA: Regeneron Genetics

BioMe BioBank Program, Mount Sinai Icahn School of Medicine	2007	82k	Mount Sinai Aims to enroll 1 Million people In a new genetic sequencing project	EHR	Self-completed questionnaires history and lifestyle	Blood	Genotyping<br>WES	NIH	• Scientific collaborator for sequencing DNA: Regeneron Genetics
**United Kingdom**									
UK Biobank	2007-2010	500k<br>40-69 y/o	100k with imaging data<br>50k exercise stress test with ECG<br>100k with activity monitor	Anthropometrics<br>Blood pressure<br>Lung vital capacity<br>Bone density<br>Intra ocular pressure	Self-completed lifestyle and general health	Blood<br>Urine	500k with microarray<br>50k WES (planned 130k 2020)<br>WGS planned	UK DoH<br>MRC<br>Wellcome Trust<br>£62M to date	• Phased releases for imaging, WES, WGS data.<br>• Primary care data, Hospital-linked admissions<br>• Cancer registry data linked<br>• Death status linked
**Canada**									
Montreal Heart Institute Biobank	2007	Target 30k<br>20k completed	--	EHR	Questionnaires updated every 4 years	Blood	Genotyping and sequencing of sub-cohorts selected for specific studies	Montreal Heart Institute Foundation	• Collaboration with AstraZeneca Biobank
**China**									
China Kadoorie Biobank	2004-2008	512k<br>30-79 y/o	Subgroup 25k tested every few years<br>Resurveys in 2008, 2013, and 2020	Baseline clinical variables	Medical and lifestyle	Blood	~100k with candidate array (384 SNPs)<br>Up to ~100k with GWAS array (700k SNPs)	Chinese Government	• Joint venture between University of Oxford and Chinese Academy of Medical Sciences<br>• 8 years of follow-up data available
China Taizhou Biobank	2004	100k	Planned	Anthropometrics<br>Tissue<br>Disease-oriented	Interviewer-conducted surveys	Blood	Unknown	Chinese Government	• Fudan University Institute of Health Sciences<br>• Includes CSF, frozen tissue, FFPE
**India**									
National Cancer Tissue Biobank**	2014	Target 25k over 5 years	--	Cancer biobank	--	Tissue samples	--	Indian Government	• Public-private partnership joint initiative of Department of Science and Technology (DST), Government of India and Indian Institute of Technology Madras (IITM), Chennai.
**The Netherlands**									
Lifelines Cohort Study	2006-2013	167k<br>25-50 y/o and 3 generations invited	Add on studies reviewed on request, e.g.	Anthropometrics<br>Blood pressure<br>ECG	Lifestyle<br>Health<br>Personality	Blood<br>Urine<br>Stool	GWAS array<br>Planned microbiome		• 30-year longitudinal study

		(i.e., offspring, parents and partners)	Omics profiling agreed in subset of 10k	Lung vital capacity<br>Cognitive	Work<br>Living environment	Scalp hair			
**Iceland**									
deCODE	1996-present	230k to date	Planned enrolment entire population of Iceland (364k)	Medical records<br>Genealogical records	Unknown	Blood	GWAS array (337k)<br>WES<br>WGS (15k)	Private initiative (Amgen)	
**Finland**									
Fingen	2018-2024	520k	No	EHR		Blood	GWAS array (520k)	Finnish Universities and private partners	• Private, includes pharmaceutical companies<br>• 230k samples collected to date
**Estonia**									
Estonian Biobank, University of Tartu	2002-present	200k	--	EHR	Self-completed questionnaire, including data about their health status, lifestyle and diet	Blood	Genome-wide genotyping arrays (200k)<br>Metabolomics (11k)<br>WGS (3k)<br>WES (2k)	National budget through the Ministry of Social Affairs	• The Estonian Biobank is one of the founding members of BBMRI-ERIC, a major European research infrastructure that brings together European biobanks

DoH=Department of Health; ECG=electrocardiograph, FFPE=formalin-fixed, paraffin-embedded; GWAS=genome wide association studies; k=Thousand; NHGRI=National Human Genome Research Institute; SNP=single nucleotide polymorphisms; WES=whole exome sequencing; WGS=whole genome sequencing; y/o=Years-old;
[*] Only the most common sample types are included in this table; information on additional samples collected is provided whenever applicable. [**] indicates limited information available.

Biobank Program at Icahn School of Medicine, NIH *All of Us*, Estonian Biobank, Auria Biobank (Finland), FinnGen (Finland), deCODE Genetics (Iceland), Biobank Graz (Austria), BioBank Japan, China Kadoorie Biobank, Pakistan Genomic Resource Biobank, Qatar Biobank
